# Non-interference of Bovine-Human reassortant pentavalent rotavirus vaccine ROTASIIL® with the immunogenicity of infant vaccines in comparison with a licensed rotavirus vaccine^☆^

**DOI:** 10.1016/j.vaccine.2018.07.064

**Published:** 2018-09-05

**Authors:** Sajjad Desai, Niraj Rathi, Anand Kawade, Padmasani Venkatramanan, Ritabrata Kundu, Sanjay K. Lalwani, A.P. Dubey, J. Venkateswara Rao, D. Narayanappa, Radha Ghildiyal, Nithya J Gogtay, P. Venugopal, Sonali Palkar, Renuka Munshi, Ashish Bavdekar, Sanjay Juvekar, Nupur Ganguly, Prabal Niyogi, Kheya Ghosh Uttam, Alpana Kondekar, Dipti Kumbhar, Smilu Mohanlal, Mukesh C. Agarwal, Parvan Shetty, Kalpana Antony, Bhagwat Gunale, Abhijeet Dharmadhikari, Jagdish Deshpande, Uma Nalavade, Deepa Sharma, Anurag Bansal, Yuxiao Tang, Jorge Flores, Prasad S. Kulkarni

**Affiliations:** a Serum Institute of India Pvt. Ltd., Pune, India; b PATH, India; c Vadu Rural Health Program KEM Hospital Research Centre Vadu, Pune, India; d Sri Ramachandra Medical Centre, Chennai, India; e Institute of Child Health, Kolkata, India; f Bharati Vidyapeeth Medical College & Hospital, Pune, India; g Maulana Azad Medical College, New Delhi, India; h Gandhi Medical College & Gandhi Hospital, Secunderabad, India; i JSS Medical College & Hospital, Mysore, India; j T.N. Medical College & B.Y.L. Nair Charitable Hospital, Mumbai, India; k Seth GS Medical College & KEM Hospital, Mumbai, India; l Andhra Medical College, Visakhapatnam, India; m Enterovirus Research Centre, Mumbai, India; n Quest Diagnostics India Private Limited, Gurgaon, India; o PATH, USA

**Keywords:** Bovine-human reassortant pentavalent rotavirus vaccine (BRV-PV), DTwP-HepB-Hib vaccine, Oral polio vaccine (OPV), Interference, Immune response

## Abstract

**Background:**

A newly developed bovine-human reassortant pentavalent vaccine (BRV-PV, ROTASIIL®) was tested for its potential effect on the immunogenicity of concomitantly administered EPI vaccines in infants in a randomized controlled study in India.

**Methods:**

In this Phase III, multicenter, open label, randomized, controlled study, three doses of BRV-PV or two doses of Rotarix® and one dose of placebo were given to healthy infants at 6, 10, and 14 weeks of age. Subjects also received three doses of DTwP-HepB-Hib (diphtheria, tetanus, whole-cell pertussis, hepatitis B, and haemophilus influenzae type b conjugate – pentavalent vaccine) and oral polio vaccine concomitantly at 6, 10, and 14 weeks of age and a single dose of inactivated polio vaccine at 14 weeks of age. Blood samples were collected four weeks after the final vaccination to assess immune responses to all the vaccines administered. For diphtheria, tetanus, hepatitis B, Hib, polio type 1, and polio type 3 antibodies, non-interference was to be supported if the lower limit of the two-sided 90% confidence interval (CI) for the seroprotection rate difference for the BRV-PV group minus the Rotarix® group was >10.0%. For pertussis antibodies, non-interference was to be supported if the lower limit of the two-sided 90% CI for the ratio of geometric mean concentrations (GMCs) was >0.5.

**Results:**

A total of 1500 infants were randomized to either BRV-PV (1125 infants) or Rotarix® (375 infants), of which 1341 completed the study as per the protocol. More than 97% of subjects achieved seroprotective antibody titres against diphtheria, tetanus, hepatitis B, Hib, polio type 1, and polio type 3 in both groups. The difference in seroprotection rates between the BRV-PV group and the Rotarix® group for all these antibodies was less than 1%. The ratio of GMCs of anti-pertussis IgG concentrations for the BRV-PV group versus Rotarix® was 1.04 [90% CI: 0.90; 1.19].

**Conclusion:**

BRV-PV does not interfere with the immunogenicity of concomitantly administered routine infants vaccines.

## 1 Introduction

Considering that rotavirus gastroenteritis is a significant public health problem, especially in low-resource countries [1], the World Health Organization (WHO) recommends universal immunization with rotavirus vaccines [2]. To meet the global demand, a bovine-human reassortant pentavalent rotavirus vaccine (BRV-PV, ROTASIIL®) was recently launched in India. The vaccine has shown satisfactory clinical performance in various studies and has an added feature of heat stability [3], [4], [5], [6].

Like other live oral rotavirus vaccines, BRV-PV is indicated for routine infant immunization with a three-dose schedule at 6, 10, and 14 weeks of age. For the last many years, vaccines against diphtheria, pertussis, tetanus, hepatitis B, haemophilus influenzae type b, and polio have also been given at these time points. As per regulatory requirements, it has to be demonstrated that any new vaccine does not interfere with the immune responses to these vaccines [7].

The present study was undertaken with two primary objectives: (a) to demonstrate clinical lot-to-lot consistency of BRV-PV and (b) to demonstrate non-interference in the immune responses of concomitant vaccines. The results of safety, rotavirus vaccine immunogenicity, and lot-to-lot consistency are under publication separately. The present paper provides the findings on the non-interference with the immunogenicity of concomitant vaccines.

## 2 Methods

### 2.1 Ethics

The study was approved by the institutional ethics committees and the Indian regulatory authorities. Parent(s) gave a written informed consent for participation of their children in the study. The study conduct was in compliance with the Declaration of Helsinki and good clinical practices guidelines. Identity of participants was always kept confidential.

### 2.2 Study design

This Phase III multicentre, open-label, randomized, controlled study was conducted between December 2015 and November 2016. The study subjects (n = 1,500) were equally randomized to four arms with 375 subjects each; three received different lots of BRV-PV and one received Rotarix®.

Three doses of BRV-PV or two doses of Rotarix® and one dose of placebo were administered at 6, 10, and 14 weeks of age. As per the Universal Immunisation Programme (UIP) in India, the subjects also received three doses each of DTwP-HepB-Hib and oral polio vaccine (OPV) at 6, 10, and 14 weeks of age. In addition, the subjects received inactivated polio vaccine (IPV) at the age of 14 weeks. Trivalent OPV (tOPV) and bivalent OPV (bOPV) were given to subjects before and after 25 April 2016, respectively, in accordance with the global switch mandated by WHO [8].

### 2.3 Selection criteria

The subjects were healthy infants of 6–8 weeks of age at the time of enrolment who had received HepB vaccine and OPV at birth. Infants with any acute disease were temporarily excluded from enrolment. Significant malnutrition or any systemic disorder, congenital abdominal disorders, intussusception, abdominal surgery, impairment of immunological function, persistent diarrhea, or allergy to any components of the study vaccines were exclusion criteria.

### 2.4 Investigational products

BRV-PV is a live attenuated, pentavalent, human-bovine reassortant rotavirus vaccine (ROTASIIL®, Serum Institute of India Pvt. Ltd., SIIPL). It is available as a lyophilized powder along with 2.5 ml buffered diluent [4]. Rotarix® (GlaxoSmithKline plc, Belgium) is also a live attenuated rotavirus vaccine. The vaccine used in the study was a lyophilized vaccine to be reconstituted with a liquid diluent in a pre-filled oral applicator.

DTwP-HepB-Hib vaccine (Pentavac® PFS, SIIPL, Batch No. 137K5001A, Expiry December 2016) was given by intramuscular injection. Each 0.5 ml dose of Pentavac® PFS contains: diphtheria toxoid ≤25 Lf (≥30 IU), tetanus toxoid ≥2.5 Lf (≥40 IU), B. pertussis (whole cell) ≤16 OU (≥4 IU), HBsAg (rDNA) ≥10 µg, and Hib polysaccharide (PRP) conjugated to tetanus toxoid 10 mcg.

tOPV (BioPolio®, Bharat Biotech International Ltd., India, Batch No. 63AS15001, Expiry August 2017) was given orally. Each dose of two drops (0.1 ml) contains not less than 10^6.0^ CCID_50_ infectious units of type 1 poliovirus, 10^5.0^ CCID_50_ infectious units of type 2 poliovirus, and 10^5.8^ CCID_50_ infectious units of type 3 poliovirus.

bOPV (BioPolio®, Bharat Biotech International Ltd., India) was also given orally. Two batches (Batch No. 68CV00416008, Expiry December 2017 and Batch No. 68CV00716014, Expiry February 2018) were used in the study. Each dose of two drops (0.1 ml) contains not less than 10^6.0^ CCID_50_ infectious units of type 1 poliovirus and 10^5.8^ CCID_50_ infectious units of type 3 poliovirus.

One dose of IPV (Poliovac PFS®, SIIPL, Batch No. 151K5006A, Expiry March 2017) was given intramuscularly. Each 0.5 ml dose contains not less than inactivated poliovirus type 1, Mahoney strain 40 D antigen units, poliovirus type 2, MEF-I strain 8 D antigen units, and poliovirus type 3, Saukett strain 32 D antigen units.

BRV-PV, Rotarix®, DTwP-HepB-Hib, and IPV vaccines were stored at 2–8 °C, while both bOPV and tOPV were stored at ≤−20 °C.

### 2.5 Randomization and blinding

The eligible subjects were randomized to three BRV-PV groups or to Rotarix® group according to a computer-generated randomization list. A block size of 12 was used to ensure a 1:1:1:1 balance for the study. While the study was not blinded to the clinical staff or the parents, the laboratory personnel were not aware of the treatment allocation.

### 2.6 Immunogenicity assessment

Four weeks after the third dose, a single blood sample was collected from each child. Polio type 1 and type 3 antibodies were tested by a validated neutralization assay at the Enterovirus Research Centre, Mumbai. Neutralizing antibody titers ≥1:8 were considered seroprotective. IgG antibodies against diphtheria, pertussis, tetanus, Hib, and hepatitis B were tested at Quest Diagnostics India Private Limited, Gurgaon. Commercial ELISA kits of Virion - Serion (diphtheria, pertussis, and tetanus), Binding site (Hib), and Vitros (hepatitis B) were used for antibody measurements. The kits used for testing were validated by the manufacturers as well as by the laboratory. Antibody concentrations ≥0.1 IU/mL (for diphtheria and tetanus), ≥1 mcg/mL (for Hib) and ≥10 mIU/mL (for hepatitis B) were considered seroprotective. Seropositivity for pertussis was defined as concentration of >50 IU/ml, as per the manufacturer’s instructions.

### 2.7 Statistical analysis

The full analysis (FA) population included all subjects in the enrolled population who were randomized and received at least one dose of study vaccination and had post-vaccination immunogenicity results. The per-protocol (PP) population included all subjects in the FA population who had received all three doses of study vaccines as per the assigned group with no major protocol violations. The PP population was the primary analysis population for all immunogenicity objectives, while the FA population results were supportive. A one-sided type I error rate of 0.05 was used for the non-inferiority comparisons. All statistical analyses were conducted using SAS® software.

Non-inferiority for polio type 1, polio type 3, diphtheria, tetanus, Hib, and hepatitis B was evaluated using the two-sided 90% CI for the difference between seroprotection rates in the combined BRV-PV lots and Rotarix® group. The two sided 90% CIs were obtained using the Newcombe hybrid score method. Non-inferiority was declared if lower limits of the two-sided 90% CI for the difference (BRV lots combined, Rotarix® reference) was >−10.0% for the above six antibodies.

Non-inferiority for pertussis was examined using the ratio of GMCs (combined BRV groups and Rotarix®) with a non-inferiority criterion that the lower limit of the two-sided 90% CI for the ratio is >0.5. The log_10_-transformed concentrations were used to construct a 90% CI for the ratio using t-distribution for the mean difference between the combined BRV and reference groups. The mean difference and the corresponding 90% CI limits were exponentiated to obtain the GMC ratio and its 90% CI.

The sample size calculation was driven by the lot-to-lot consistency objective, which required an evaluable sample size of 900 subjects for the BRV-PV lots combined and 300 subjects for the Rotarix® group. At this size, this study provided >99.9% power for testing potential interference for all of the UIP vaccines.

## 3 Results

A total of 1585 subjects were screened and 1500 eligible subjects were randomized. Of these, 1374 subjects completed the study and were part of the FA population. Of these, 33 subjects had a major protocol violation; therefore, 1341 subjects were part of the PP population. The details of the exclusions and the demographic data are given in the lot-to-lot consistency publication (Under publication). The results from the PP population are presented.

### 3.1 Data on vaccines used

84 (5.6%) subjects received only one dose, 19 (1.27%) received two doses, and 1394 (92.93%) received all three doses of rotavirus vaccines. All subjects received DTwP-HepB-Hib and OPV concomitantly. All subjects also received IPV at the age of 14 weeks.

24.73% of subjects received tOPV, 42.93% received bOPV, and 32.13% received a combination of tOPV and bOPV concomitantly with BRV-PV and Rotarix®/placebo doses.

### 3.2 Seroprotection/seropositivity results

UIP vaccines elicited high immunogenicity with most of the subjects achieving seroprotective antibody levels. More than 97% of the subjects presented seroprotective antibody titres against Hib in both groups. For all other antigens, seroprotection was seen in >99% subjects in both groups (Table 1).

**Table 1:** Seroprotection/seropositivity rates for UIP vaccines 4 weeks following three vaccine doses (PP Population).

Antibody	BRV-PV	Rotarix®	Difference (BRV-PV – Rotarix®)
	n/N*, % (95% CI)	n/N, % (95% CI)	%, (90% CI)
Poliovirus 1	1010/1010, 100% (99.64 – 100)	328/330, 99.39% (97.83–99.93)	0.61% (0.12–1.81)
Poliovirus 3	1006/1010, 99.60% (98.99–99.89)	328/330, 99.39% (97.83–99.93)	0.21% (−0.42–1.44)
Diphtheria	999/1008, 99.11% (98.31–99.59)	328/329, 99.70% (98.32–99.99)	−0.59% (−1.27–0.52)
Pertussis**	666/1008, 66.07% (63.06–68.99)	217/329, 65.96% (60.56–71.07)	0.11 (−4.73–5.14)
Tetanus	1008/1008, 100% (99.63–100)	329/329, 100% (98.89–100)	0.00% (−0.27–0.82)
Hepatitis b	999/1002, 99.70% (99.13–99.94)	324/327, 99.08% (97.34–99.81)	0.62% (−0.09–1.99)
Hib	987/1008, 97.92% (96.83–98.71)	321/329, 97.57% (95.27–98.94)	0.35% (−1.03–2.28)

95% CI = 95% exact (Clopper-Pearson) confidence interval.

90% CI = 90% Newcombe score confidence interval.

* n = seroprotected/seropositive; N = Number of subjects for whom immunological result was available.

** Seropositivity defined as antibody concentration > 50 IU/ml.

The difference in seroprotection rates for different antibodies between BRV-PV and Rotarix® groups ranged from −0.59 [90% CI: −1.27; 0.52] to 0.62 [90% CI: −0.09; 1.99]. Non-inferiority was demonstrated, as the lower limit of the two-sided 90% CI for the rate difference for all the antibodies was >−10.0% (Table 1).

For Hib, 100% short term seroprotection (≥0.15 mcg/mL) was seen in both treatment groups. Pertussis seropositivity rates were similar (around 66%) in both the groups, with a difference of 0.11% [CI: −4.73; 5.14] (Table 1).

The percentage of subjects with polio-seroprotective titers according to type of OPV received was also assessed. For both type 1 and type 3 antibodies, seroprotection rates were more than 98% in both the groups, irrespective of whether they received tOPV alone, bOPV alone or mixed schedules (Table 3). Thus, BRV-PV did not interfere with OPV, irrespective of the type of OPV received (bOPV or tOPV).

### 3.3 GMC/GMT results

The ratio of anti-pertussis GMC for the combined BRV-PV/Rotarix® group was 1.04 [90% CI: 0.90; 1.19] (Table 2). Thus, the lower limit of the 90% CI for the GMCs ratio was >0.5, which supports the non-inferiority hypothesis that the BRV-PV vaccine does not interfere with pertussis vaccination. For all other antibodies too, the GMC ratios for the combined BRV-PV/Rotarix® group were around 1.

**Table 2:** Comparison of GMCs/GMTs 4 weeks post-final vaccination for UIP vaccines (PP Population).

Antibody		BRV-PV		Rotarix®	Ratio (BRV-PV/Rotarix®)
	N	GMC, (95% CI)	N	GMC, (95% CI)	Value (90% CI)
Poliovirus type 1	1010	932.78 (895.76–971.32)	330	966.24 (888.58–1050.68)	0.97 (0.89–1.04)
Poliovirus type 3	1010	564.28 (531.99–598.54)	330	564.74 (508.95–626.64)	1.00 (0.90–1.10)
Diphtheria	1008	1.07 (1.03–1.12)	329	1.18 (1.11–1.26)	0.90 (0.85–0.96)
Pertussis	1008	67.43 (62.21–73.10)	329	64.94 (55.68–75.74)	1.04 (0.90–1.19)
Tetanus	1008	1.30 (1.26–1.35)	329	1.35 (1.26–1.44)	0.97 (0.91–1.02)
Hepatitis B	1002	652.66 (621.82–685.04)	327	698.05 (636.59–765.44)	0.93 (0.86–1.02)
Hib	1008	7.51 (7.26–7.77)	329	7.56 (7.11–8.04)	0.99 (0.94–1.05)

N = Number of subjects for whom immunological result was available.

95%/90% CI = asymptotic standardized 95%/90% confidence interval.

**Table 3:** Comparison of polio GMTs as per the type of OPV received (PP Population).

	N	BRV-PV GMC, (95% CI)	N	Rotarix® GMC, (95% CI)	GMC ratio (90% CI)
*Polio Type 1 antibody*					
All doses tOPV	214	741.88 (660.85–832.84)	65	782.97 (632.24–969.63)	0.95 (0.78–1.16)
All doses bOPV	443	1067.97 (1003.91–1136.12)	154	1084.18 (951.19–1235.76)	0.99 (0.87–1.11)
Two doses tOPV + one dose bOPV	183	797.52 (712.37–892.85)	62	809.73 (606.79–1080.56)	0.98 (0.76–1.28)
One dose tOPV + two dose bOPV	170	926.03 (842.04–1018.40)	49	874.39 (727.37–1051.13)	1.06 (0.89–1.25)

*Polio Type 3 antibody*					
All doses tOPV	214	485.09 (431.24–545.66)	65	464.31 (385.87–558.71)	1.04 (0.86–1.27)
All doses bOPV	443	647.30 (589.55–710.70)	154	690.13 (597.00–797.78)	0.94 (0.81–1.09)
Two doses tOPV + one dose bOPV	183	511.02 (450.09–580.19)	62	477.07 (363.34–626.42)	1.07 (0.83–1.38)
One dose tOPV + two dose bOPV	170	536.94 (467.06–617.29)	49	491.90 (366.12–660.90)	1.09 (0.85–1.41)

N = Number of subjects for whom immunological result was available.

95%/90% CI = asymptotic standardized 95%/90% confidence interval.

Though statistically not compared, the GMTs for both type 1 and type 3 polioviruses were higher with bOPV alone as compared to tOPV alone in both the groups.

## 4 Discussion

In this Phase III study we evaluated the potential interference of SIIPL's BRV-PV with routine UIP vaccinations in healthy infants. We found that all UIP vaccines elicited high immunogenicity in the great majority of the subjects. BRV-PV did not interfere with the immunogenicity of any of the antigens, as assessed by both seroprotection rates and GMT ratios.

No interference by parenteral vaccines like DTP, HepB and Hib has been observed in several trials of Rotarix [9], [10], [11], RotaTeq [12], [13] and Rotashield, [14]. Further, co-administration of Rotarix with conjugate pneumococcal vaccine have also shown no interference [11]. Several studies have pointed out to some interference of OPV with the responses to rotavirus vaccines, mostly after the first dose [15]. Subsequent doses have resulted in no significant changes in the overall responses to the rotavirus component and clinical protection has been maintained [9], [10], [16].

No study has detected interference by rotavirus vaccine on the seroconversion or seroprotection of OPV [13]. A booster dose of Rotarix given simultaneously with measles-rubella vaccine did not have an impact on the immunogenicity of any of the two vaccines (measles or rubella) [17].

As per their summary of product characteristics [18], [19] and the studies quoted above, Rotarix and Rotateq do not interfere with the immune response of DTPa, HepB, IPV, Hib, pneumococcal conjugate vaccine, meningococcal serogroup C conjugate vaccine and OPV.

A similar phase III study in India assessed whether ROTAVAC® interfered with the immune response to OPV and DTwP-HepB-Hib vaccines. Like ours, that study found no interference with the immune response to those vaccines [20].

It should be noted, that in our study, both OPV and IPV were received by all children since mid-way through the study, since the Ministry of Health, Government of India switched tOPV to bOPV and IPV was used to fill the gap between the two vaccines. Though we did not find any interference by BRV-PV with polio vaccines irrespective of the type of OPV used, the switch gave us an opportunity to compare immunogenicity of tOPV, bOPV and mixed schedules. The seroprotection rates were high (>98%) with both the vaccines but the GMTs appeared lower with tOPV as compared to bOPV. Similarly, a study in India had found lower GMTs as well as lower seroconversion rates with tOPV as compared to bOPV [21].

We used Rotarix as a control, rather than placebo because it is not ethically possible to keep a group of infants which receives no rotavirus vaccine, especially because WHO recommends rotavirus vaccination for all the infants [2] and because India has included rotavirus vaccination in the UIP [22].

All participants had received a birth dose of OPV as it is part of the UIP in India. They also received three doses OPV (initially trivalent and bivalent after April 2016) and one dose of IPV during the study. This was as per WHO’s polio endgame strategy which included a global switch from tOPV to bOPV and addition of a single dose of IPV in EPI schedules in countries using OPV [23].

Our results are thus in line with studies of all previously licensed rotavirus vaccines. To conclude, BRV-PV does not interfere with immune response of UIP vaccines.

## Conflict of interest

Dr. Prasad S. Kulkarni, Dr. Sajjad Desai, Dr. Bhagwat Gunale, and Mr. AbhijeetDharmadhikari are employed by SIIPL, which manufactures the BRV-PV.

## Funding

The study was funded by PATH and SIIPL.
